# Discovery and Characterization of a Novel Ampelovirus on Firespike

**DOI:** 10.3390/v12121452

**Published:** 2020-12-16

**Authors:** Yaqin Wang, Yu Song, Yongzhi Wang, Mengji Cao, Tao Hu, Xueping Zhou

**Affiliations:** 1 State Key Laboratory of Rice Biology, Institute of Biotechnology, Zhejiang University, Hangzhou 310058, China; yaqinwang@zju.edu.cn (Y.W.); 21816171@zju.edu.cn (Y.S.); 21916185@zju.edu.cn (Y.W.); 2 State Key Laboratory for Biology of Plant Diseases and Insect Pests, Institute of Plant Protection, Chinese Academy of Agricultural Sciences, Beijing 100193, China; 3 National Citrus Engineering and Technology Research Center, Citrus Research Institute, Southwest University, Chongqing 400712, China; caomengji@cric.cn

**Keywords:** *Ampelovirus*, firespike leafroll-associated virus, *Closteroviridae*, infectious cDNA clone

## Abstract

A novel RNA virus was identified in firespike (*Odontonema tubaeforme*) plants exhibiting leaf curling and chlorosis. The molecular features of the viral genomic RNA and proteins resemble those of ampeloviruses. Based on sequence comparisons and phylogenetic analysis, we propose a new species in the genus *Ampelovirus*, which we have tentatively named Firespike leafroll-associated virus (FLRaV). Bioassays showed that the virus is mechanically transmissible to *Nicotiana benthamiana*. In addition, a full-length cDNA clone of FLRaV could successfully infect *N. benthamiana* via agroinfiltration.

## 1. Introduction

Members of the genus *Ampelovirus* (family *Closteroviridae*) are plant RNA viruses that infect mainly fruit crops in orchards and stock nurseries, causing serious reductions in fruit yield and quality worldwide [1]. Ampeloviruses contain large monopartite positive-sense, single-stranded RNA genomes, ranging from 13,071 nucleotides (nt) in Pineapple mealybug wilt-associated virus 1 (PMWaV-1) to 18,659 nt in Grapevine leafroll-associated virus 1 (GLRaV-1) [2,3,4,5], which can be transmitted by mealybugs and soft scale insects, or through vegetative propagation [6]. The filamentous virions are non-enveloped, ∼2000 nm in length and 12 nm in diameter [7,8].

Typically, the genome of ampeloviruses contains two major characteristic gene modules [6,7]. The replication gene block (RGB), located towards the 5′ end of the viral genome, consists of open reading frames (ORFs) 1a and 1b. ORF1a encodes a polyprotein, while ORF1b encodes an RNA–dependent RNA polymerase (RdRp) [9,10,11,12,13]. Five ORFs downstream of the RGB form the quintuple gene block (QGB), sequentially encoding for a putative small hydrophobic protein, a heat shock protein 70 homologue (HSP70h), a putative protein with a conserved domain of heat shock protein 90 homologue, a coat protein (CP), and a minor coat protein (CPm) [14,15]. According to genome size, gene organization and sequence similarity, ampeloviruses can be further clustered into two subgroups. For members in subgroup I, a large, GC-rich intergenic region (above 600 nt) is located between two gene blocks, and towards the 3′ end of the viral genome, a variable array of genes encoding structural and accessory proteins can be found [16,17,18,19]. Members in subgroup II have reduced genome size and complexity; the intergenic region between the two gene modules is relatively small (~150 nt), and no variable coding region has been identified at the 3′ end of the viral genome [20,21,22,23].

In this paper, we identified a novel virus from the common ornamental shrub Firespike (*Odontonema tubaeforme*) by high-throughput sequencing of ribo-depleted total RNA. The virus showed the typical molecular and biological properties of members belonging to the genus *Ampelovirus*.

## 2. Materials and Methods

### 2.1. Plant Materials

Leaf samples showing curling and chlorosis symptoms were collected from firespike plants (*Odontonema tubaeforme*) in 2017 in Chenshan Botanical Garden, Shanghai, China. *Nicotiana benthamiana*, *N. glutinosa*, *N. tabacum*, *N. rustica*, and *N. sylvestris* were grown on soil at 25 °C with a 14:10 h photoperiod.

### 2.2. RNA Extraction and Next-Generation Sequencing Analysis

Total RNA was extracted from collected firespike leaves by using RNAiso Plus (TaKaRa, Tokyo, Japan) as per the manufacturer’s instructions. The cDNA library was prepared using the TruSeq RNA Sample Prep Kit v2 (Illumina, San Diego, CA, USA) after removing ribosomal RNA with the Ribo-ZeroTM rRNA Removal Kit (Epicentre, Madison, WI, USA). Paired-end sequencing was then performed on an Illumina NovaSeq 6000 (HaploX Biotechnology, Jiangxi, China). Clean reads were assembled using StringTie Software and contigs greater than 50 nt were then analyzed by BLASTx at a threshold E-score cutoff of 10^−5^.

### 2.3. Determination of the 5′ and 3′ End of Genomic RNAs

The exact 5′ and 3′ end sequences of viral genomic RNA were determined by rapid amplification of cDNA ends (RACE) [24]. Briefly, the first strand cDNA was synthesized using M-MLV reverse transcriptase (TaKaRa, Tokyo, Japan) with gene-specific primers (Table S1). The RNA template was removed with Ribonuclease H (RNase H) (Thermo Fisher Scientific, Waltham, MA, USA). The 3′ end of the cDNA was attached to either a poly(A) or poly(C) tail by terminal deoxynucleotidyl transferase (TdT) (TaKaRa). Nested PCR was carried out with combinations of gene-specific primers and adaptor primers (Table S1). PCR products were ligated into the pClone007 vector (TSINGKE, Beijing, China) and sequenced.

### 2.4. Virus Genome Sequence Analysis

ORFs were predicted and analyzed using SnapGene^®^. Transmembrane helices were predicted using TMHMM 2.0 (http://www.cbs.dtu.dk/services/TMHMM/). Amino acid sequence alignments were performed using Cluster W by MegAlin^®^ (DNAStar, Madison, WI, USA) and MUSCLE by MEGA X. Phylogenetic analyses were performed using the maximum likelihood method by MEGA X. The GenBank accession numbers of sequences analyzed in the study are listed in Table S2.

### 2.5. Mechanical Inoculation

One gram of symptomatic firespike leaves was ground in 0.1 M phosphate buffer (pH 7.2) with 2% Celite. Two leaves of *N. benthamiana, N. glutinosa*, *N. tabacum*, *N. rustica* or *N. sylvestris* plants at the 4-leaf-stage were gently rubbed with the extracted leaf sap. Plants were then maintained in a growth chamber at 25 °C with a 14:10 h photoperiod.

### 2.6. Construction of the FLRaV Infectious cDNA Clone

The FLRaV infectious cDNA clone was generated as described [25,26]. Briefly, the FLRaV genome was divided into eleven fragments arbitrarily, and amplified with the appropriate primer pairs. A total of 5 µg of DNA containing equal molar ratios of viral cDNA fragments and 1.8 µg of linearized pCB301 vector were co-transformed into yeast strain Gold according to the Yeastmaker Yeast Transformation System 2 (Clontech, Tokyo, Japan). The transformants were plated on a synthetic agar medium and grown at 30 °C for 3 days. The recombined plasmids were extracted using the Qiagen plasmid midi kit (Qiagen, Hilden, Germany) and sequenced. The recombinant pCB301-FLRaV binary plasmid was transformed into *A. tumefaciens* strain EHA105. Equal volumes of *Agrobacterium* cultures harboring pCB301-FLRaV as well as constructs expressing two RNA silencing suppressors, barley stripe mosaic virus (BSMV) γb and tomato bushy stunt virus (TBSV) p19, were mixed and co-infiltrated into four-week-old *N. benthamiana* leaves [26,27,28]. The plants were then maintained in a growth chamber at 25 °C with a 14:10 h photoperiod.

## 3. Results

### 3.1. Identification of FLRaV through Next-Generation Sequencing

**Figure 1:**
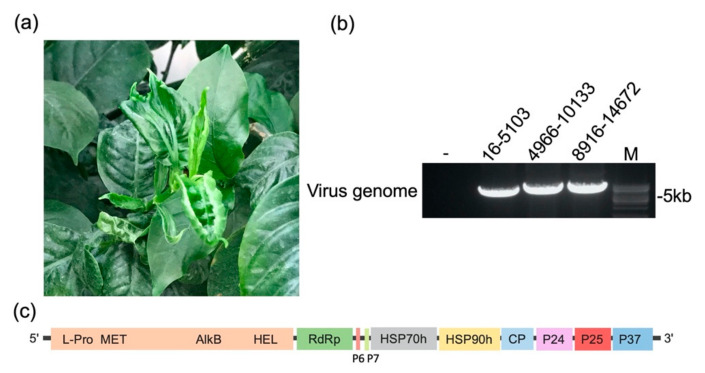
Features of firespike leafroll-associated virus. (**a**) Curly leaf and chlorosis symptoms of Firespike leafroll-associated virus (FLRaV) in infected firespike; (**b**) RT-PCR analysis of three sections of the FLRaV RNA genome on symptomatic firespike leaves—indicates RT-PCR reaction without primers; (**c**) Schematic representation of the genome organization of FLRaV. Boxes indicate the position of different open reading frames with their respective products, whereas black lines depict untranslated genomic regions. L-PRO, MET, AlkB, HEL: protease, methyltransferase, AlkB and helicase domains of the replicase polyprotein; RdRp: RNA-depenent RNA polymerase; p6: 6 kDa protein; p7: 5 kDa protein; HSP70h: heat shock protein 70 homology; HSP90h: heat shock protein 90 homology; CP: coat protein; p24: 21 kDa protein; p25: 25 kDa protein; p37: 37 kDa protein.

Next-generation sequencing was performed on a cDNA library prepared from ribo-depleted RNA from firespike leaves with severe upward rolling and chlorosis symptoms (Figure 1a). A total of 60,446,396 high quality reads were obtained and subjected to de novo assembly. BLASTx analyses of the identified contigs in the National Center for Biotechnology Information (NCBI) database showed that 3916 clean reads (68 reads per million reads mapped) shared significant nucleotide similarities with the genomic sequence of viruses in the genus *Ampelovirus,* yielding an average coverage of the whole genome at 40.08. Additional reads with similarities to the cucumber mosaic virus and columnea latent viroid were identified in the BLASTx analysis (Table S3). Only sequences relevant to the potential ampeloviruses were analyzed in the present study.

To determine the complete sequence of the in silico identified viral RNA, reverse transcription-PCR (RT-PCR) was performed on total RNA extracted from the infected firespike leaves. Sixteen sets of primer pairs were designed based on the contigs identified to yield amplicons of ~800 bp in size with ~100 bp overlapping regions. The RT-PCR products were sequenced and assembled into a contiguous sequence. After that, the exact 5′ and 3′ ends of the viral RNA genome were determined by rapid amplification of the cDNA ends (RACE). The presence of the RNA genome of the virus in the symptomatic samples was further confirmed by RT-PCR within virus-specific primer sets (Figure 1b). Based on these findings, the virus was provisionally named Firespike leafroll-associated virus (FLRaV).

### 3.2. Complete Sequence and Organization of FLRaV Genome

**Figure 2:**
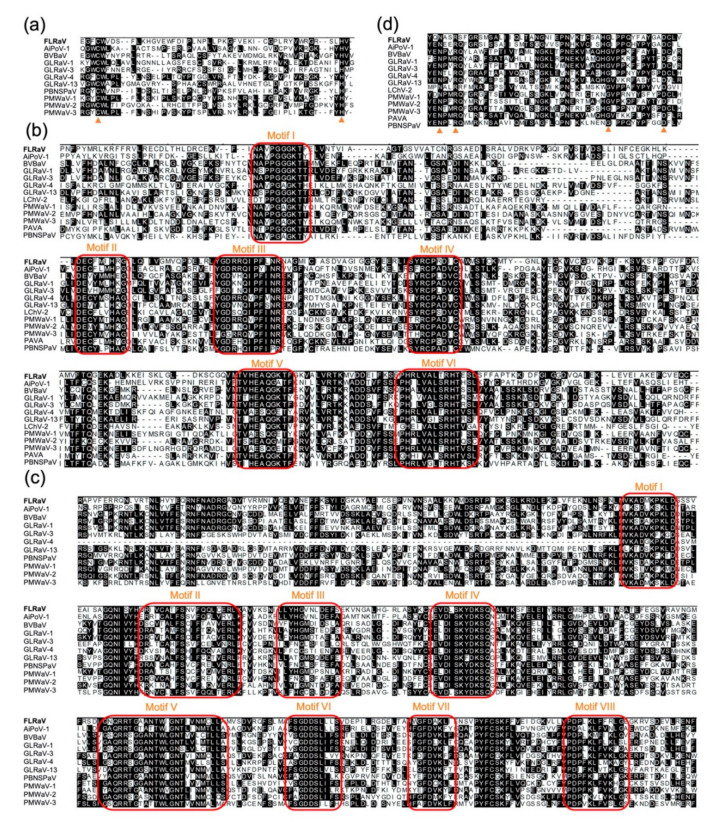
Multiple alignments of FLRaV open reading frames (ORFs) with other ampeloviruses. (**a**) Multiple alignments of ORF1a L-PRO of FLRaV and other ampeloviruses. Triangles indicate the conserved catalytic cysteine and histidine residues. (**b**) Multiple alignments of ORF1a HEL of FLRaV and other ampeloviruses. Red boxes indicate six conserved motifs. (**c**) Multiple alignments of RdRp of FLRaV and other ampeloviruses. Red boxes indicate eight conserved motifs. (**d**) Multiple alignments of CP of FLRaV and other ampeloviruses. Triangles indicate four N, R, G and D residues of the filamentous plant virus coat protein.

The complete genome of the identified virus is 14,672 nt in length and possesses nine potential ORFs (Figure 1c). The genome organization and characteristics resemble those of members of the genus *Ampelovirus* [2,7,8]. The FLRaV genome contains two major characteristic gene modules. The replication gene module occupies the bulk of the FLRaV virus genome. ORF 1a codes for a putative 2005 aa-long replication-associated polyprotein. The L-PRO domain with conserved catalytic cysteine (C^208^) and histidine (H^251^) residues was identified by its homology with L-PRO domains of other ampeloviruses (Figure 2a) [9]. A methyltransferase domain (Pfam 01660) in position aa 337–697 and an AlkB domain (Pfam 03171) in position aa 1702–1972 were identified downstream of the L-PRO domain [10,11]. The *C*-terminal region of ORF1a contained a HEL domain (Pfam 01443); the amino acid residues spanning six conserved HEL motifs of FLRaV were identical to the same regions in other ampeloviruses (Figure 2b) [12]. ORF1b of FLRaV encodes a putative 501 amino acid polypeptide with a predicted molecular mass of 56.8 kDa, identified as the RdRp (pfam 00978), containing eight conserved motifs of RdRps of positive-stranded RNA viruses (Figure 2c) [13]. ORF2 and 3 code for two small proteins of 56 and 60 aa, with predicted molecular masses of 6.4 and 6.8 kDa, respectively. No apparent homologs were found for these two proteins, while in silico analysis revealed that both proteins have a putative transmembrane helix. Amino acid sequence alignments of ORF3 with ORFs of other members of the genus *Ampelovirus* with similar positions within the genome showed that all the proteins have a conserved hydrophobic region (Figure S1). Putative proteins encoded by ORF4 and ORF5 are 534 aa (59.2 kDa) and 532 aa (61.1 kDa), containing a cellular molecular chaperone HSP70 (HSP70h) domain (pfam 00012) and a HSP90 (HSP90h) domain (pfam 03225) [14]. ORF6 encodes a protein of 306 aa (33.5 kDa), predicted to be the capsid protein (CP), and contains a sequence domain characteristic of CPs encoded by filamentous plant viruses (Figure 2d) [15]. ORF7 codes for an uncharacterized polypeptide with a molecular weight of 24.7 kDa. No canonical motifs were identified in this protein. The five ORFs 3–7 comprise the conserved quintuple gene module. ORF8 is located downstream of the QGB and encodes a polypeptide with a molecular weight of 25.0 kDa. This protein has no statistically significant similarity with any proteins in the available databases; however, we detected a viral nucleic acid binding domain (Pfam: 05515) common to positive-strand RNA viruses, and two Clathrin-binding boxes of Aftiphilin (Pfam: 15045), which is involved in vesicle trafficking. The 3′-proximal ORF encodes a protein with a molecular mass of 37.0 kDa. Amino acid sequence analysis showed that this protein has high identity with members of the Myb family of transcription factors. The function of the proteins encoded by ORF8 and 9 remains elusive, although by analogy to similarly located ORFs of other members of the family *Closteroviridae*, these two proteins could be involved in virus movement and suppression of host RNA interference. The intergenic region between the two gene modules is 149 nt in length, and the 5′ untranslated region (UTR) and 3′ UTR of FLRaV are 244 and 153 nt in length, respectively. Sequence comparison analysis with other ampeloviruses showed that these two regions share the highest sequence similarities (77.6% and 73.7%) with the 5′ UTR of Air potato ampelovirus-1 (AiPoV-1) and Grapevine leafroll-associated ampelovirus-4 (GLRaV-4), respectively [22,23].

### 3.3. Phylogenetic Relationship of FLRaV with Other Ampeloviruses

**Figure 3:**
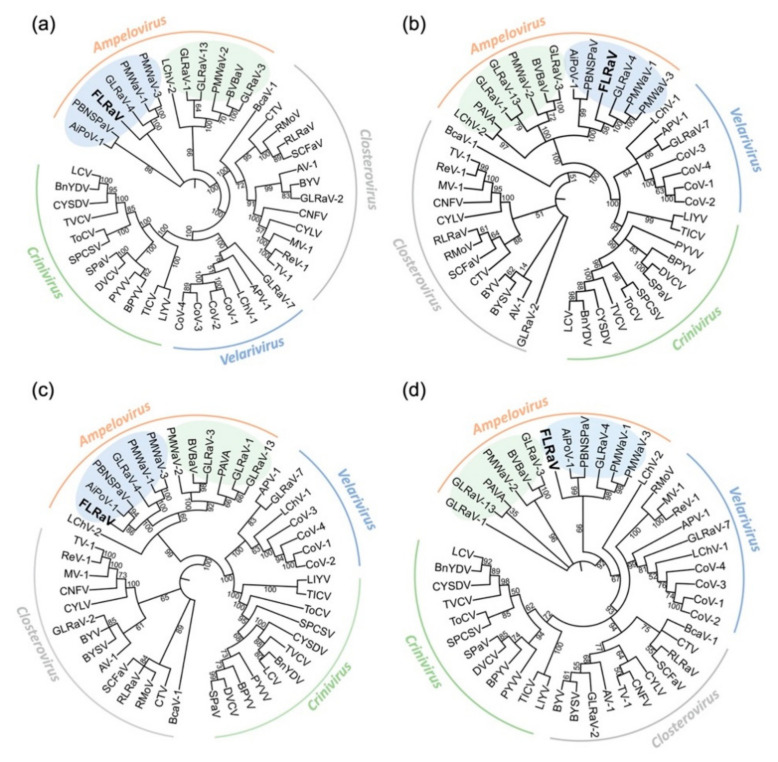
Phylogenetic trees constructed using the amino acid sequences of ORF1a (**a**), RdRp (**b**), HSP70h (**c**), and CP (**d**) of members in the family *Closteroviridae*. Bootstrap values (%) for 1000 replicates are indicated. Virus names are shown in Table S2. The light green and light blue ovals represent subgroups I and II of the *Ampelovirus*, respectively.

Amino acid sequence comparisons of four taxonomically relevant gene products, ORF1a, HSP70h, RdRp, and CP proteins of FLRaV, with homologous *Ampelovirus* species showed a range of 22.5–50.6% sequence identity (Table 1). These sequence differences surpass the 25% threshold of genetic variability within the genus [29]. Phylogenetic analyses, based on the amino acid sequences of ORF1a, HSP70h, RdRp, and CP of closteroviruses, were further performed using the maximum likelihood algorithm. The trees showed that FLRaV always clustered with other ampeloviruses, confirming its position within the genus *Ampelovirus* (Figure 3). In addition, it was noticed that all four phylogenetic trees placed FLRaV in the subgroup II clade of the genus on a branch with AiPoV-1, GLRaV-4, PMWaV-1, PMWaV-3, and Plum bark necrosis stem pitting-associated ampelovirus (PBNSPaV) (Figure 3). Consistent with the phylogenetic analysis, the FLRaV genome possesses typical traits of the *Ampelovirus* subgroup II, including reduced genome size and a small intergenic region between the two gene modules. Taken together, these results clearly indicate that FLRaV represents a distinct species within the GENUS *Ampelovirus*.

### 3.4. Infectivity of FLRaV on Nicotiana benthamiana

**Figure 4:**
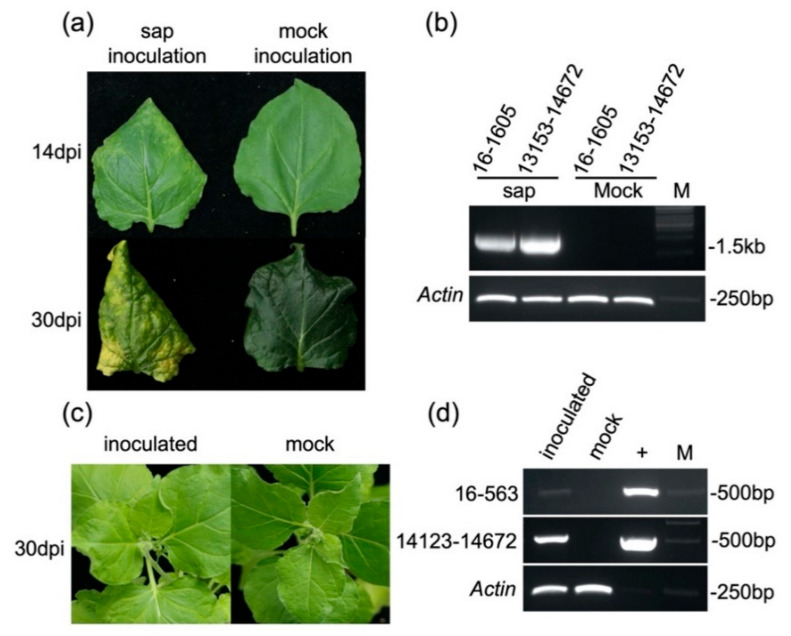
Symptoms on leaves of *Nicotiana benthamiana* infected by FLRaV: (**a**) Curly leaves and chlorotic spots on the upper leaves of *N. benthamiana* infected by sap inoculation at 14 and 30 dpi, respectively; (**b**) RT-PCR detection of FLRaV in upper leaves of inoculated *N. benthamiana* plants with two different primer pairs specific for two regions of the FLRaV genome indicated above the lanes. Samples were taken at 30 dpi. (**c**) No visible symptoms are observed on the upper leaves of full-length FLRaV cDNA-inoculated *N. benthamiana* plants at 30 dpi; (**d**) RT-PCR detection of FLRaV in upper leaves of inoculated *N. benthamiana* plants with two different primer pairs specific for two regions of the FLRaV genome. Samples were taken at 30 dpi. Recombined plasmid containing the FLRaV cDNA serves as a positive control.

In order to test whether FLRaV could be transmitted to other herbaceous hosts, sap from symptomatic firespike was mechanically inoculated into five different varieties of tobacco plants, including *N. benthamiana*, *N. glutinosa*, *N. tabacum*, *N. rustica*, and *N. sylvestris*. At 30 days post inoculation, only *N. benthamiana* plants displayed virus symptoms including leaf curling and chlorosis (Figure 4a and Table 2). In accordance with this, the FLRaV genome was detected by RT-PCR with specific primers in the upper leaves of infected *N. benthamiana* (Figure 4b and Figure S2a). These results indicated that FLRaV could be readily mechanically transmitted from its host plant to *N. benthamiana*.

To further determine the infectivity of FLRaV in *N. benthamiana*, the full-length cDNA clone of FLRaV was generated and inserted into an *Agrobacterium* binary expression vector [25]. A culture of *A. tumefaciens* cells harboring the FLRaV full-length cDNA clone was infiltrated into *N. benthamiana* leaves. At 30 days post inoculation, the presence of the virus could be detected by RT-PCR on the upper leaves of about 81.3% (26/32) of inoculated plants (Figure S2b). However, unlike mechanically inoculated *N. benthamiana* plants, no visible symptoms were observed in the infiltrated *N. benthamiana* plants (Figure 4c,d). These results suggested that the FLRaV full-length cDNA clone could replicate and move in *N. benthamiana* without inducing any typical viral symptoms.

## 4. Discussion

Based on the characterization of the FLRaV genome, we found that this virus possesses several features of the genus *Ampelovirus*. (i) The virus contains a large, single-stranded, positive-sense RNA genome (Figure 1); (ii) The ORF1a (Pro-L, MTR, AlkB, HEL), RdRp and CP contain conserved motifs identical to the orthologous proteins of members within the genus (Figure 2); (iii) Phylogenetic analyses of ORF1a, RdRp, HSP70h and CP placed FLRaV in the same clade with members of the genus *Ampelovirus* (Figure 3). In addition, pairwise identity scores of ORF1a, RdRp, HSP70h, and CP with other ampeloviruses are no more than 51% (Table 1), suggesting that FLRaV identified in the firespike plant appears to be a novel virus species in the genus *Ampelovirus*.

Although clustered in the subgroup II of *Ampelovirus* through phylogenetic analysis, the FLRaV virus genome possesses some features similar to members of subgroup I. For example, unlike members of subgroup II, the FLRaV virus genome has a small ORF positioned between the RGB and the QGB, and two additional ORFs located at the 3′ end of the genome. To the best of our knowledge, it is the first identified ampelovirus that possesses features of both subgroups. By analogy to similarly located ORFs of other members of the family *Closteroviridae* (e.g., P19.7 of GLRaV-3, P24 of GLRaV-2, P20 of PMWaV-2), FLRaV P25 and P37 might be involved in the suppression of host RNA interference [30,31,32]. Through amino acid sequence analysis, a viral nucleic acid binding domain and two clathrin-binding boxes of aftiphilin were identified in P25, suggesting that this protein might function in virus movement. It is worthwhile to further investigate whether P25 facilitates virus trafficking in plants.

It has been shown that GLRaV-2 in the genus *Closterovirus* is mechanically transmissible to the model plant *N. benthamiana* [33,34], but no ampelovirus has displayed this feature so far. Through vector–mediated transmission, GLRaV-3 in the genus *Ampelovirus* was successfully transmitted to *N. benthamiana*, although the transmission rates were very low and the best transmission rates were obtained using transgenic *N. benthamiana* plants expressing HC-Pro from the turnip mosaic virus [35]. Here, we reported the first ampelovirus for successful mechanical sap transmission from symptomatic firespike to *N. benthamiana*. It should be noted that reads with similarity to cucumber mosaic virus (CMV) and columnea latent viroid were identified in the BLASTx analysis as well, and firespike is a common host of CMV [36]. Further study will be required to determine whether co-infection of CMV or CLVD could help in FLRaV transmission and symptom manifestation. Full-length cDNA inoculation of FLRaV could successfully infect *N. benthamiana* without inducing visible symptoms, similar to the full-length cDNA clone of GLRaV-3 [26]. Future work will focus on whether FLRaV full-length cDNA clones could be used as an efficient virus-based vector system for expressing reporter genes in *N. benthamiana*.

## 5. Conclusions

By using unbiased high-throughput sequencing and conventional molecular biology tools, a new ampelovirus, tentatively named FLRaV, was identified. Full-length cDNA of FLRaV was infectious to a convenient laboratory plant host *N. benthamiana.* This experimental system can be used for further analysis of ampelovirus-host interactions.

## Figures and Tables

**Table 1:** Amino acid sequence identity between proteins encoded by FLRaV and other ampeloviruses.

	Amino Acid (%)			
	ORF 1a	RdRp	HSP70h	CP
AiPoV-1 ^a^	28.6	43.7	50.4	35.9
BVBaV	25.2	35.1	35.4	26.5
GLRaV-1	28.2	36.6	35.5	24.1
GLRaV-3	29.8	37.9	37.8	28.7
GLRaV-4	31.2	44.7	50.0	42.4
GLRaV-13	27.6	42.7	37.5	22.5
PBNSPaV	30.6	44.5	50.6	34.8
PMWaV-1	29.9	40.4	47.8	36.6
PMWaV-2	24.9	35.6	35.0	27.3
PMWaV-3	29.5	41.4	47.1	43.8
LChV-2	26.4	35.8	37.4	28.9
PAVA	- ^b^	39.4	33.4	26.7

^a^ Virus names are shown in Table S2 ^b^ Only a partial sequence of PAVA ORF1a is available in the GenBank database.

**Table 2:** RT-PCR detection of FLRaV in five different plant species mechanically inoculated with sap form symptomatic firespike.

Plant Species	No. of Positive Plants/Total
*N. benthamiana*	27/32
*N. glutinosa*	0/8
*N. tabacum*	0/9
*N. rustica*	0/9
*N. sylvestris*	0/9

## Supplementary Materials

The following are available online at https://www.mdpi.com/1999-4915/12/12/1452/s1, Figure S1. Multiple alignments of ORF3 of FLRaV. Multiple alignments of ORF3 of FLRaV and ORFs of other members of the genus *Ampelovirus* with similar positions within the genome. Hydrophobic amino acids were indicated with yellow background. Figure S2. RT-PCR analysis of FLRaV RNA genome. (a) RT-PCR detecting FLRaV RNA genome on upper leaves of 32 inoculated *N. benthamiana* at 30 dpi; (b) RT-PCR detecting FLRaV RNA genome on the upper leaves of 32 full-length cDNA inoculated *N. benthamiana* at 30 dpi. Table S1. Primers used in this study. Table S2. The GenBank accession numbers of sequences used in the study. Table S3. Coverage of cucumber mosaic virus (CMV) and columnea latent viroid genome sequences in the next-generation sequencing datasets.
