# Supplementary figures and images for: Discovery and Characterization of a Novel Ampelovirus on Firespike
Source: Viruses. 2020 Dec 16;12(12):1452. doi: 10.3390/v12121452 (PMC7766885; doi:10.3390/v12121452)

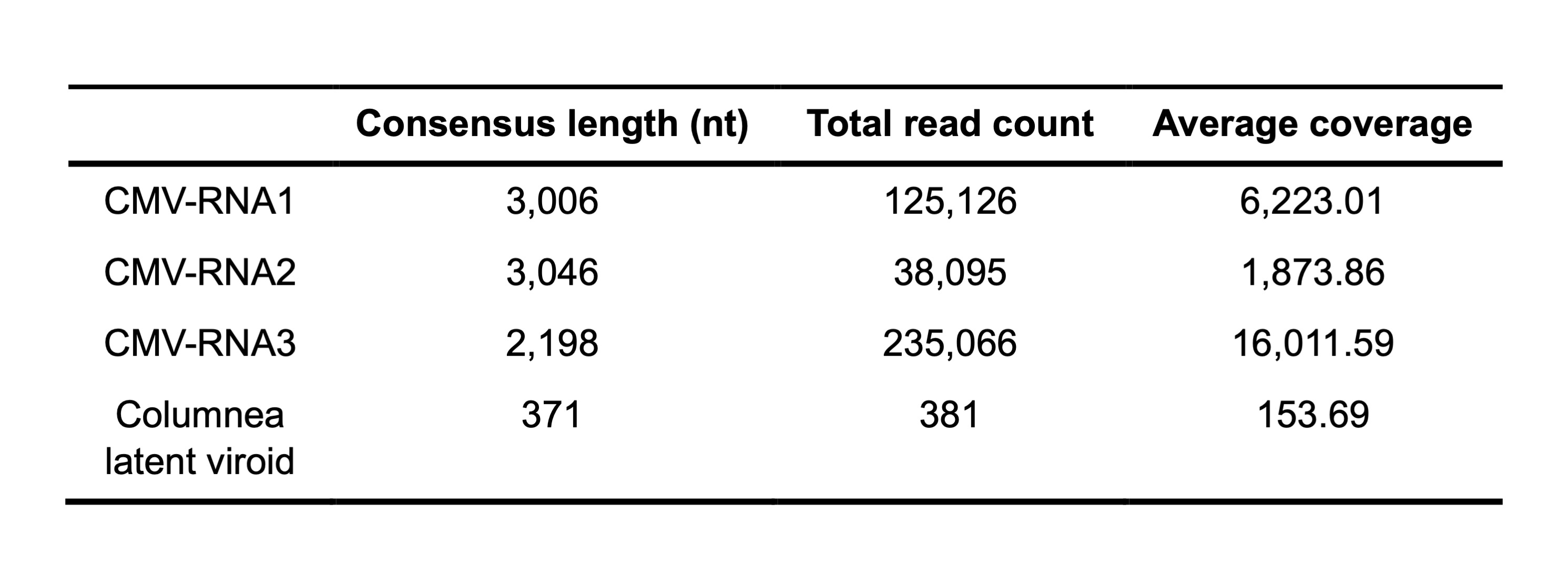

Supplement: Supplementary file 1 [file viruses-12-01452-s001.zip › Supplementary Material/Table S1.jpg]

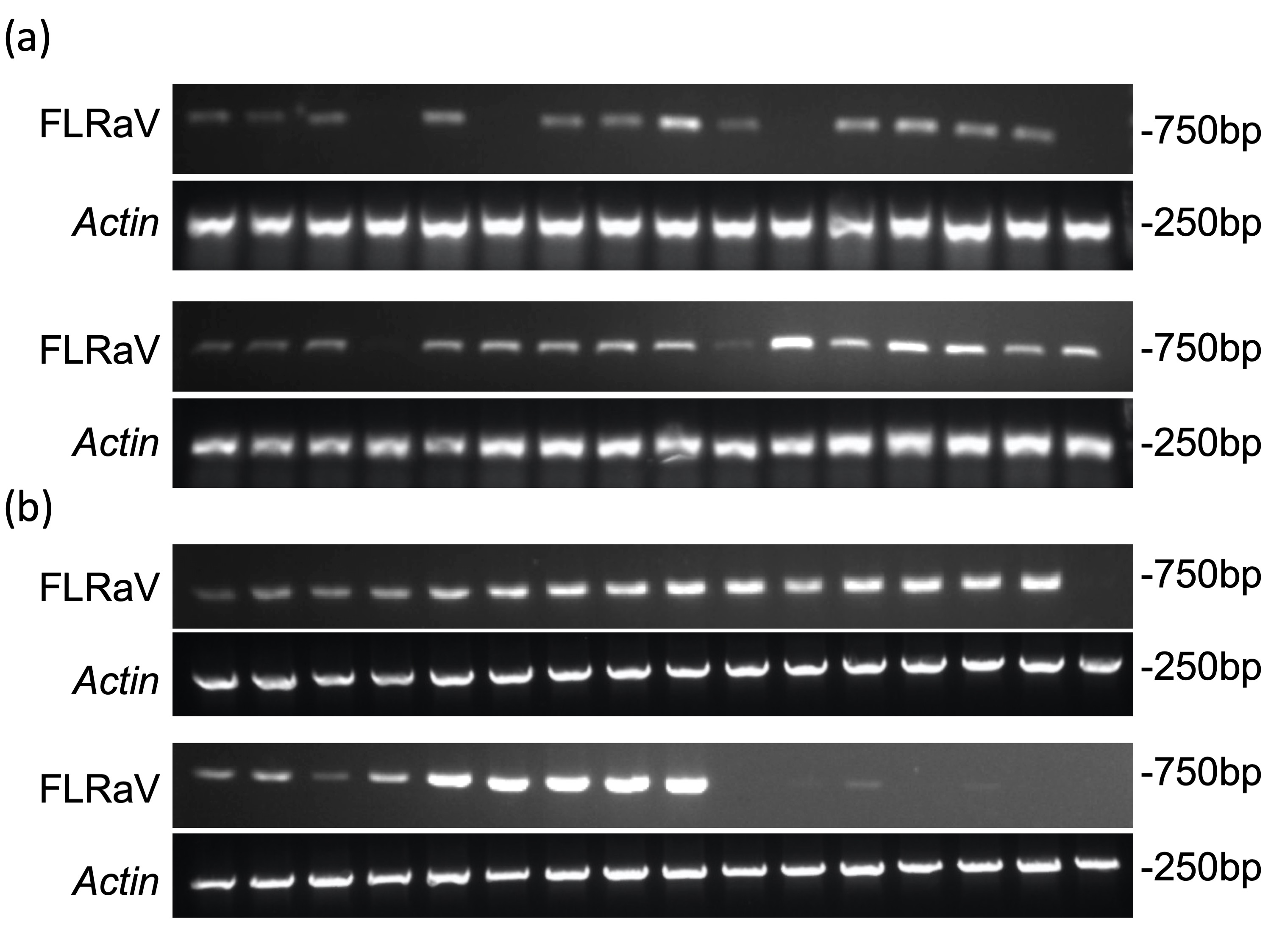

Supplement: Supplementary file 1 [file viruses-12-01452-s001.zip › Supplementary Material/Figure S3.jpg]

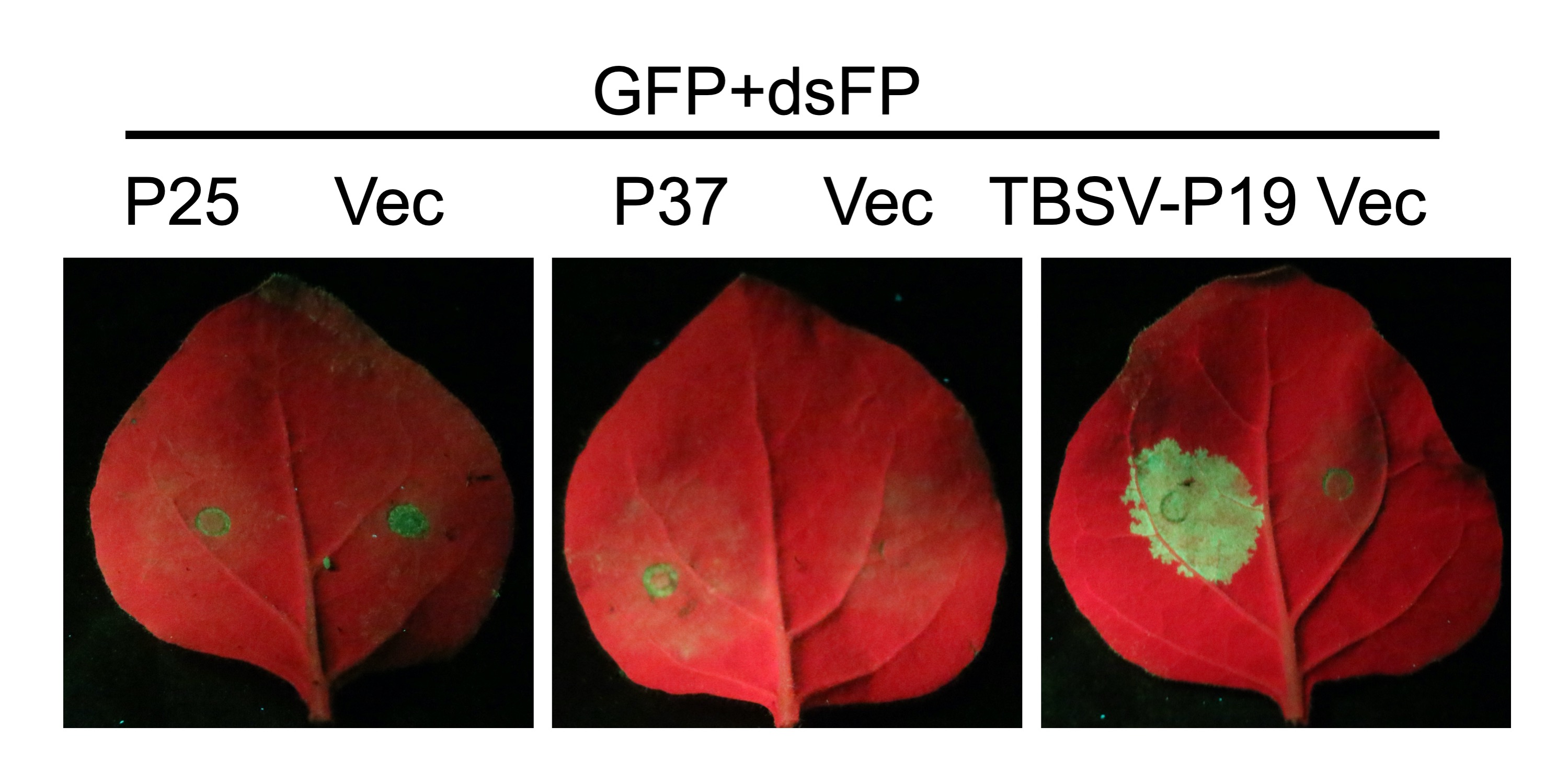

Supplement: Supplementary file 1 [file viruses-12-01452-s001.zip › Supplementary Material/Figure S2.jpg]

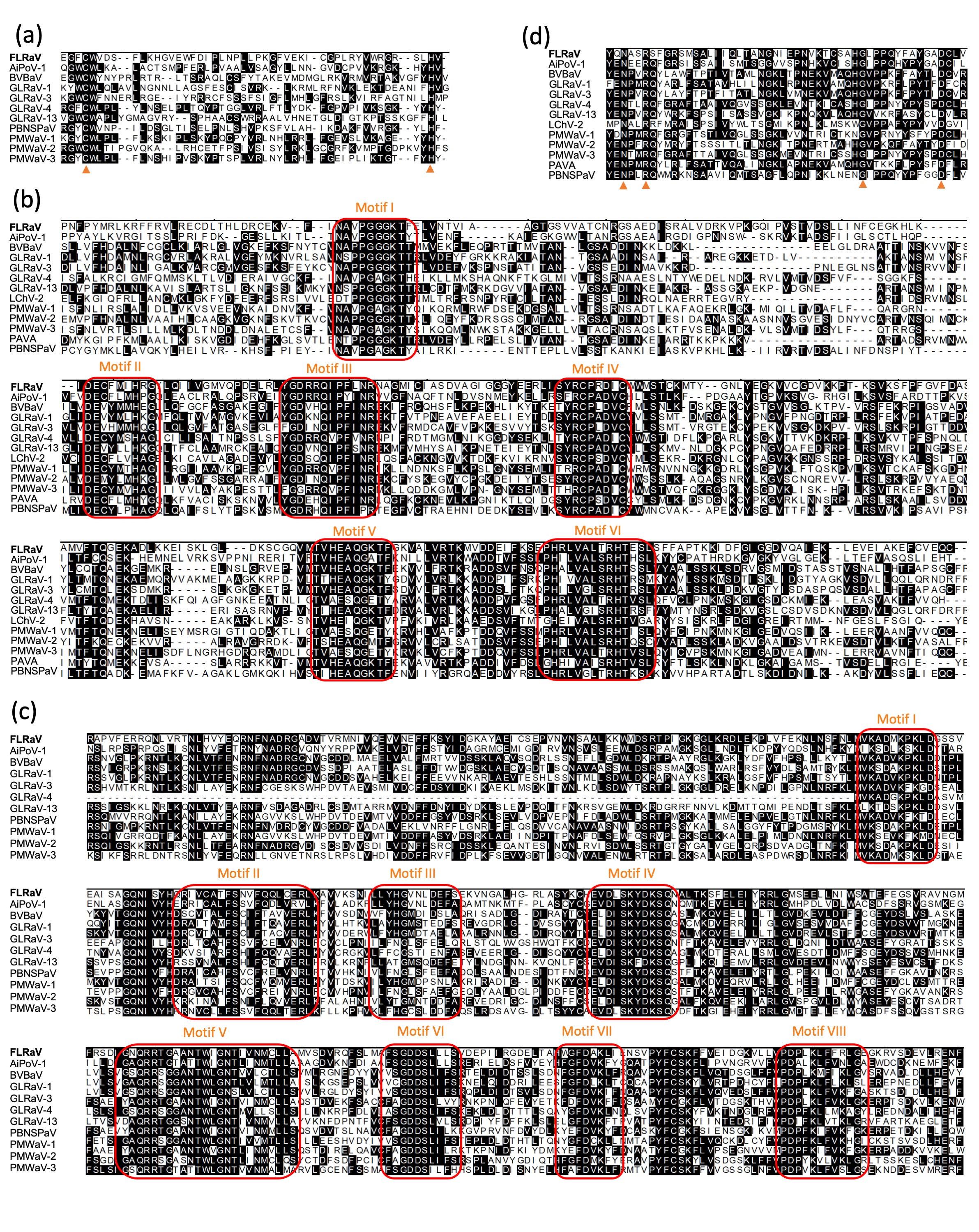

Supplement: Supplementary file 1 [file viruses-12-01452-s001.zip › Supplementary Material/Figure S1.jpg]
